# Birth weight discordance, gene expression, and DNA methylation: A scoping review of epigenetic twin studies

**DOI:** 10.1371/journal.pone.0315549

**Published:** 2025-11-19

**Authors:** Dany Laure Wadji, Zsofia Nemoda, Chantal Martin-Soelch, Linda Booij, Chantal Wicky

**Affiliations:** 1 Department of Educational and Counselling Psychology, Faculty of Education, McGill University, Montreal, Canada; 2 Department of Psychology, University of Fribourg, Fribourg, Switzerland; 3 Department of Health Sciences, Université du Québec à Rimouski, Rimouski, Canada; 4 Eating Disorders Continuum and Douglas Research Centre, Douglas Mental Health University Institute, Montreal, Canada; 5 Department of Molecular Biology, Semmelweis University, Budapest, Hungary; 6 CHU Sainte-Justine Azrieli Research Center, Montreal, Canada; 7 Department of Psychiatry, McGill University, Montreal, Canada; 8 Department of Biology, University of Fribourg, Fribourg, Switzerland; University of Connecticut, UNITED STATES OF AMERICA

## Abstract

**Background:**

Birth weight is considered as an important indicator of environmental conditions during prenatal development. Molecular mechanisms, including epigenetic modifications, play central roles in the body’s adaptation to ever-changing environmental conditions. Twin study designs offer a powerful approach for distinguishing environmental from genetic effects. Specifically, within-pair comparisons of monozygotic twins can be used to differentiate unique individual environmental factors from shared environmental and genetic contributions. Notably, numerous studies in monozygotic twins have shown associations between prenatal environment and birth weight discordance (BWD), and suggested a potential involvement of gene expression and epigenetic factors mediating the association.

**Objective:**

To conduct a scoping review of the literature on definitions of BWD and on epigenetic modifications and gene expression changes associated with BWD in twins.

**Method:**

Following PRISMA guidelines, we searched PubMed and Ovid MEDLINE(R) databases and included 34 twin studies focusing on birth weight and epigenetic or gene expression outcomes.

**Results:**

There is a lack of consensus on BWD values when comparing groups of twins for their risks of perinatal mortality and morbidity, which vary between 15–30% depending on the type of placentation and gestational age. The gene expression twin studies measured mostly metabolism-related candidate genes in placental tissues. Only small-scale twin studies measured BWD associated with gene expression patterns on genome-wide level using neonatal cells. Most DNA methylation twin studies conducted epigenome-level analyses, and studies differ substantially in terms of tissue type and age of the children. Differences in DNA methylation patterns measured in blood or saliva samples of the twins later in life were mostly in genes related to signal transduction, cell differentiation and proliferation processes.

**Conclusion:**

Transcriptional changes of placental glucose transporters and hypoxia-induced proteins possibly reflect compensatory processes in twin pregnancies. Gene ontology analysis of the differentially methylated genes associated with BWD pointed to transcription regulation and tissue development.

## Introduction

The proportion of multiple births (i.e., giving birth to two or more infants) has increased worldwide with an average of 16.8 per 1000 women giving birth to twins [1], possibly due to the increased use of assisted reproductive technologies, such as *in vitro fertilization* [2,3]. Multiple birth situation is likely to affect birth weight, as babies are prone to be born prematurely or before 37 weeks [4]. Birth weight is considered as an important indicator of environmental conditions during prenatal development [5,6]. Birth weight discordance (BWD), i.e., substantial difference in birth weight between newborns, occurs in 10–29% of twin pregnancies [7,8]. This variability reflects differing measurement approaches, including percentage thresholds (e.g., ≥ 15% or ≥ 20%), absolute weight differences, and gestational age adjustments. Nonetheless, greater BWD has been associated with risk for adverse outcome, such as preterm birth [9], cumulative morbidity, neurodevelopmental impairment in the twin with the lower weight [8,10], intraventricular hemorrhage and cerebral palsy in the larger twin [8,11], and intrauterine or perinatal death [12,13]. BWD is therefore of great importance for obstetrical risk assessment and for adjusting the prenatal management of such pregnancies.

Numerous twin studies have assessed the underlying molecular mechanisms of BWD (e.g., studying gene expression or DNA methylation differences in twins samples). However, we are not aware of a recent synthesis of this literature. Therefore, the primary aim of the present review was to synthesize results of studies that examined the association between BWD, epigenetic factors and/or gene expression in twins. However, before presenting the findings from twin studies analyzing molecular mechanisms, it is essential to first consider the broader context of how birth weight may reflect underlying epigenetic changes, the methodological contributions of twin study designs in disentangling genetic and environmental influences, the key biological and clinical factors associated with BWD, and its varying threshold definitions. Therefore, we will discuss these points before reviewing the research on BWD, DNA methylation and gene expression.

### Birth weight and epigenetic markers

Molecular mechanisms, including epigenetic modifications play an important role in the body’s dynamic adaptation to environmental conditions during life, starting after conception, at implantation [14]. Given that birth weight is considered as a proxy of prenatal development and is also indicative of adult health outcomes [14], epigenetic mechanisms, such as DNA methylation and histone modifications, might provide explanations on how adverse prenatal environment is associated with low birth weight [15], and how birth weight may in turn affect health later in life [16]. Recent findings suggest interindividual differences of DNA methylation patterns in humans at certain gene regions potentially reflect distinct environmental effects [17]. Therefore, epigenetic patterns associated with an indicator of growth may serve as biomarkers of adverse prenatal environment [18]. Given that epigenetic modifications may be reversible, understanding these molecular mechanisms associated with birth weight could be a relevant therapeutic target that can help to minimize the long-term consequences of adverse prenatal environment.

The associations between birth weight and epigenetic markers, like DNA methylation patterns have been previously examined in several studies with singletons [15,19]. The most frequently measured marker in the human genome is the methylation of the cytosine base in a CpG dinucleotide (the letter “p” represents the phosphodiester bond between cytosine and guanine). Recently, patterns of additional covalent modifications, such as DNA hydroxymethylation, have been also analysed. Hydroxymethylcytosine is thought to be an important intermediate in active demethylation processes catalyzed by the ten-eleven translocation (TET) dioxygenase enzymes [20].

A large-scale meta-analysis of epigenome-wide association studies (EWAS) conducted on 24 birth cohorts with a total number of 8,825 neonates (singletons) showed that DNA methylation level at 1,029 CpG sites in 807 genes was associated with birth weight, in both positive and negative directions (45% and 55% of the sites, respectively) [18]. After removing unreliably associated sites, 914 loci from 729 genes were selected for further analyses. The largest positive association was found between DNA methylation at cg06378491 in the gene body of *MAP4K2* (coding for mitogen-activated protein kinase activating kinase enzyme, which can be triggered by tumor necrosis factor alpha), for each 10% increase in methylation at this site the birth weight was 178 grams higher. The CpG site with the largest negative association was cg10073091 in the gene body of the *DHCR24* oxidoreductase (catalyzing the reduction of the delta-24 double bond at cholesterol biosynthesis), which showed a 183 gram decrease in birth weight per 10% increase in methylation. Interestingly, 55 out of 914 differentially methylated sites were previously related to maternal smoking. An important methodological point in this meta-analysis was that problematic pregnancies (e.g., mothers with preeclampsia or diabetes, multiple births, and preterm births) were excluded, possibly limiting the generalizability of the findings to any type of pregnancy. Overall, the accumulated results from singletons’ pregnancies support a framework that intrauterine environmental factors induce epigenetic alterations, which influence fetal growth and hence are associated with birth weight. These epigenetic alterations can be long-lasting, but still reversible during an individual’s lifetime; therefore, the authors analyzed three age groups separately. The DNA methylation data of blood samples taken in childhood (2–13 years; n = 2,756 from 10 studies) and adolescence (16–18 years; n = 2,906 from 6 studies) pointed to a few steadily affected sites, for examples in genes coding for the *GLI2* zinc finger protein and the *HOXC4* homeobox transcription factor [18]. However, these birth weight associations were not detected in the adult samples (30–45 years; n = 1,616 from 3 studies).

**Fig 1:**
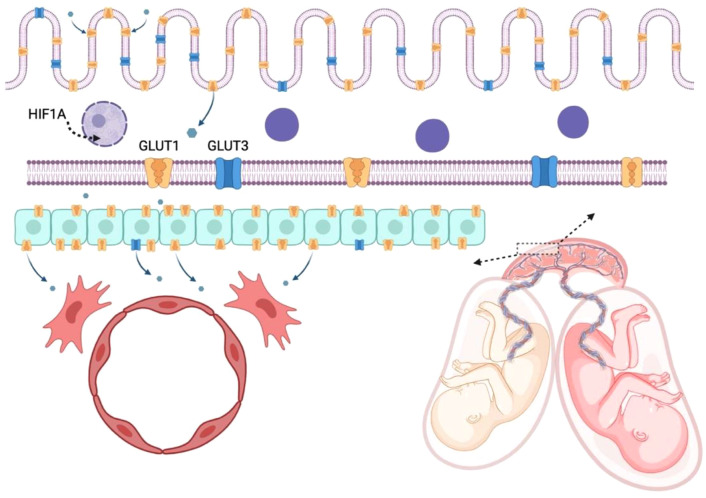
Effects of unequal nutrient supply in monozygotic twins (Created in https://BioRender.com). Although monozygotic twins have almost the same DNA sequence, there are many molecular regulatory processes differentially affected by certain in utero environmental factors, e.g., unequal blood, nutrient and oxygen supply, resulting in disparity in fetal growth. The placental section of the twin with fetal growth restriction (FGR, on the left) can have different DNA methylation and gene expression patterns compared to the placental part of the twin appropriate for gestational age (shown on the right). Hypoxia-induced changes include increased expression of glucose transporters (mostly GLUT1, GLUT3). The mechanism of glucose transport through the placental layers is highlighted in the top, hypoxia-induced changes are shown on the left side. The glucose molecule (shown as blue hexagon) is transported mainly through GLUT1 (orange channels) and GLUT3 (~30%, represented as blue channels) at the microvillous membrane of the multinucleated syncytiotrophoblasts (the outer, purple cell layer) covering the villi from the maternal side, then through the basal membrane facing the fetal side, where GLUT3 can be also present. The GLUT1:GLUT3 ratio is different at the two membranes, and also changes throughout pregnancy, depending on the blood (and nutrient supply) of the placenta, as GLUT3 enables efficient glucose transport under low extracellular glucose concentration. Glucose is transported mainly by GLUT1 across the other cell layers, i.e., cytotrophoblasts (turquoise cell layer) and endothelial cells (red colored cells in the fetal vessel), while other glucose transporters (GLUT3, GLUT4, GLUT12) are also expressed. Hypoxia-induced HIF1A leads to transcriptional changes, including increased miR-210-3p expression, which contributes to the impaired proliferation of the trophoblasts.

To study DNA methylation changes over time, the Avon Longitudinal Study of Parents and Children (ALSPAC) in the UK analyzed DNA methylation levels from 974 children at 3 timepoints (cord blood at birth, peripheral blood at age 7 and 15–17). Epigenetic research on a subsample of ALSPAC, the so-called Accessible Resource for Integrated Epigenomic Studies (ARIES) identified that birth weight was associated with increased DNA methylation level at 23 CpG sites in 14 genes, but these associations could not be detected at age 7 and 17 [21]. However, several associations of developmentally important genes, including the nuclear factor 1 X-type and the lymphotoxin alpha cytokine, could be found with later developmental phenotypes during adolescence, e.g., with weight, height, bone and fat mass. In a more recent study, DNA methylation levels measured in children’s blood samples were associated at two CpG sites with infant rapid weight gain, a risk for obesity, using an independent cohort of 104 children from the Southampton Women’s Survey as a replication sample [16]. Taken together, the results of singleton studies suggest that prenatal environment can affect fetal growth and birth weight through epigenetic mechanisms, and epigenetic markers may reflect risk for long-term health problems. However, the essential question remains of how to differentiate genetic effects from environmental effects in a sample of singleton pregnancies with substantial genetic heterogeneity. Unlike singletons, twins develop in a shared intrauterine environment with unique factors such as placental sharing and inter-twin competition, which may generate distinct epigenetic patterns (summarized at Fig 1). A twin study design can be used to identify the role of unique environmental contributions (e.g., due to unequal supply of nutrients and oxygen) while controlling for genetic variation and shared *in utero* environment (e.g., maternal diseases, smoking or alcohol use) [22]. Understanding epigenetic modifications related to birth weight is important, as these processes are modifiable and can potentially help designing future studies focusing on the intervention of long-term adverse health outcomes.

### The contribution of twin study designs to understanding BWD and epigenetic mechanisms

Twin study designs offer a unique possibility to study phenotypic variance in complex inheritance traits and diseases, because monozygotic (MZ) twins share almost identical genetic information, whereas dizygotic (DZ) twins share about 50% of genetic variants [23]. Although twins share many environmental factors before birth, such as those connected to maternal habits [24], there are certain *in utero* factors, such as placental sharing and velamentous cord insertion, which create unequal food supply for the embryos, and can be related to BWD within a MZ twin pair [25]. Twin studies are also valuable in testing postnatal environmental effects, if a proportion of twins are exposed to different physical, chemical or social environments. Therefore, twin study designs can be used to test various hypotheses within key theoretical frameworks, such as the Developmental Origins of Health and Disease (DOHAD) theory. This theory posits that exposure to prenatal and early-life events during critical periods of development impacts health outcomes and risk to disease later in life [26]. Furthermore, it has been postulated that epigenetic processes can account for how these early life factors impact long-term health outcome [27]. Over the years, many studies have examined associations between gene expression or DNA methylation patterns and BWD using twin study designs. Here, we summarize and synthesize the state of research about epigenetic differences associated with BWD in twins. Although BWD can be analyzed as a continuous variable within twin pair comparisons, many epigenetic studies have categorized twin pairs into groups (e.g., low vs. high BWD) based on threshold values. However, the methods used to calculate and define these thresholds vary widely across studies, some using absolute weight differences, others relying on percentage differences relative to the heavier twin. This variability complicates comparisons across findings and may influence the interpretation of associations with epigenetic outcomes. Importantly, several prenatal factors have been associated with BWD. These factors are particularly relevant as they may contribute not only to fetal growth discrepancies but also to downstream biological changes detectable at the molecular level. Therefore, in the present review, we will begin by examining the biological factors associated with BWD, then we will examine how the most frequently cited epidemiological twin studies have defined and operationalized BWD thresholds. We then summarize current research linking BWD to gene expression and DNA (hydroxy)methylation patterns, highlighting methodological differences and emerging findings across twin cohorts.

### Factors associated with birth weight discordance and threshold values

Substantial difference in birth weight of twins is argued to be related to prenatal factors acting asymmetrically on the two embryos, affecting the intrauterine growth of one of them while the growth of the larger twin is apparently not affected [28]. Depending on the type of placentation, i.e., monochorionic (MC) or dichorionic (DC) pregnancies, several intrinsic and extrinsic factors have been associated with BWD [13]. Intrinsic factors can be attributable to the twin pregnancy situation or to the twins themselves. For example, unequal placental sharing [29], velamentous or other abnormal cord insertion [30], and twin-twin transfusion syndrome (TTTS), i.e., when blood moves from one twin to the other, can lead to fetal growth restriction (FGR) in MC twin pregnancies [9,28]. The TTTS happens approximately in 15% of the MC twin pregnancies, when the placenta cannot provide enough oxygen and nutrients for both fetuses, and subsequently blood flows from one of the twins to the other (creating donor and recipient twin). The ultrasound signs therefore polyhydramnios in the recipient twin (due to increased blood flow, and subsequent urination into the amniotic fluid) and oligohydramnios in the donor twin, who grows slower, because of less blood flow [31]. Umbilical blood vessel abnormalities can cause growth retardation even is DC pregnancies (see for examples, [32; 33]). Hormone imbalances of the developing embryos, such as lower cord-blood adiponectin concentration can also result in growth restriction in discordant twins [34].

We have to note that certain maternal medical conditions, such as high blood pressure, preeclampsia, hepatitis C infection, and delayed childbearing impact both twins negatively [13,35,36]. These conditions, together with socioeconomical status of the family, reflected by parental education [37], or maternal habits, e.g., smoking during pregnancy are important to document when comparing groups of twins, and should be used as covariates [35].

The discordance of birth weights (in %) is calculated as = (weight of larger twin – weight of smaller twin)/ weight of larger twin × 100. The BWD thresholds are used for the prediction of perinatal mortality and morbidity. However, there is still no consensus among researchers and clinicians on BWD thresholds that predict adverse outcomes, which have been ranging between 15–40% depending on type of twins, type of placentation (chorionicity), and sex of twins (see Table 1). Important technical differences come from the study design: population-based studies with more than thousands of twin pairs used only birth and death certificates and did not have data on zygosity and chorionicity, therefore, specific subsets were analyzed separately (see for example [38]). Also, the threshold values of gestational age ranged widely, most studies included pregnancies after 24 weeks of gestation, but a few selected only term pregnancies [e.g., 39], or excluded fetal death cases [9]. Therefore, the inconsistent results about threshold of BWD may be due to that most research groups have relied basically on the level at which adverse perinatal and postnatal outcomes are more likely to occur in their specific subset of twin pregnancies. Furthermore, the included studies assessed BWD using varying methodological approaches—either as a continuous variable to capture subtle variations in risk, often through techniques like spline regression or receiver operating characteristic (ROC) curve analysis, or as a categorical variable, grouping twins based on predefined discordance thresholds. However, most studies employed a categorical analysis of BWD and the most commonly used cutoffs ranged from 15% to 30%, though specific thresholds varied depending on chorionicity, gestational age, and clinical outcomes of interest.

**Table 1:** Threshold values for birth weight discordance used in epidemiological twin studies.

Authors, birth cohort	Sample characteristics, exclusion criteria	Covariates (infant, maternal and pregnancy related variables)	BWD measure		Outcome variables associated with BWD
			BWD operationalization	BWD cutoff	
Ye et al. (2022) Foshan, China, 2012-2018	1,946 DC and 402 MC twin pairs without TTTS (total 2,348 pairs), GA ≥ 26 weeks	GA, SGA, sex, BW; maternal age, ethnicity, marital status; chorionicity, use of assisted reproductive technology, parity, obstetric complications, mode of delivery	Categorical variable with thresholds of ≥15, 20, 25, 30%; and as a continuous variable using restrictive cubic spline models and ROC curve analysis	15-30% depending on the variable	BWD ≥ 15% was associated with NICU admission (OR = 1.54; 95% CI: 1.23 - 1.93), a BWD ≥ 20% for NRDS (OR = 2.20; 95% CI: 1.38 - 3.51) and a BWD ≥ 30% for ventilator support (OR = 2.39; 95% CI: 1.25 - 4.57).
Chen et al. (2020) US, 1995-2000	98,588 sex-discordant (hence DZ) twin pairs	GA; maternal age, race, marital status, education, parity, smoking during pregnancy, pregnancy-associated hypertension	Categorical variable grouped into predefined thresholds (<10%, 10, 15, 20, 25, 30, 40, 50%, and ≥60%)	40%	BWD ≥ 40% was associated with composite adverse neonatal outcome combining NRDS, meconium aspiration syndrome, asphyxia at delivery, neonatal seizures (OR = 1.6; 95% CI: 1.1 - 2.3) and fetal death at GA 33-34 gestational weeks (OR = 17.3; 95% CI: 9.2 - 32.7).
Boghossian et al. (2019) US, 1994-2011	8,114 preterm twins with very low birth weight (401-1500 g) or gestational age 22–28 weeks, TTTS twins were excluded if it was the cause of death	GA, SGA, sex; birth year, study center; maternal ethnicity/race, antenatal steroid use	Categorical variable grouped into predefined thresholds (≤14%, >14–20%, >20–30%, and >30%)	30% (separately for smaller and larger twins)	In smaller twins: BWD > 30% was associated with increased risk for hospital death (OR = 3.2; 95% CI: 2.1-4.8), retinopathy (OR = 2.4; 95% CI: 1.1 - 5.4), bronchopulmonary dysplasia (OR = 2.3; 95% CI: 1.6 - 3.6), necrotizing enterocolitis (OR = 2.1; 95% CI: 1.3 - 3.4). In larger twins: BWD > 30% was associated with risk for patent ductus arteriosus (OR = 1.5; 95% CI: 1.1 - 1.9) and OR ~ 5 for later neurodevelopmental impairment, blindness and cerebral palsy (assessed at age 1 year).
Kim et al. (2019) US, 2012-2014	27,276 twin pairs, if twin A was delivered vaginally in cephalic presentation at GA between 36-40 weeks	Sex; maternal age, race/ethnicity, marital status, SES, obesity; parity, breech delivery of the second twin	Categorical variable grouped into predefined thresholds (0–20%, 20.01–25%, 25.01–30%, and 30.01–60%)	20%	BWD > 20% was associated with composite adverse neonatal outcome (e.g., 5-minute Apgar < 7, NICU admission, mechanical ventilation > 6 hours, neonatal seizure) (adjusted OR: 1.46; 95% CI: 1.29 - 1.65).
Vedel et al. (2017) Denmark, 2004-2008	2,378 DC and 355 MC twin pairs without TTTS (total N = 2,733 pairs), GA ≥ 24 weeks	GA, SGA, sex	Categorical variable grouped into predefined thresholds (<75th, 75th–90th, >90th percentile)	15%	BWD > 15% was associated with induced preterm delivery at GA < 34 weeks (OR = 1.71; 95% CI: 1.11 - 2.65); increased risk of infant mortality after 28 days (OR = 4.69; 95% CI: 1.07 - 20.45); risk for admission to NICU for more than a week (OR = 1.53; 95% CI: 1.21 - 1.95).
Breathnach et al. (2011) Ireland, 2007-2009	789 DC and 188 MC (14 with TTTS, total N = 963 pairs without TTTS), GA ≥ 24 weeks	GA, SGA, sex, chorionicity, birth order	Categorical variable grouped into predefined thresholds (10%, 15%, 18%, etc)	18%	BWD > 18% was associated with adverse perinatal outcome in MC twin pairs without TTTS (hazard ratio = 2.6; 95% CI: 1.6 - 4.3) and in DC twin pairs (hazard ratio = 2.2; 95% CI: 1.6 - 2.9).
Amaru et al. (2004)* New York, US, 1992-2001	1,318 twin pairs (68% had chorionicity data, TTTS is not mentioned), GA ≥ 24 weeks	GA, chorionicity, oligohydramnios (sign of TTTS), antenatal steroid use, preeclampsia	Categorical variable dichotomized into discordant vs. non-discordant	20%	BWD > 20% was associated with cesarean delivery (OR = 1.87; 95% CI: 1.22 - 2.87), adverse neonatal outcomes, like very low birth weight (OR = 8.73; 95% CI: 3.15 - 24.19), NICU admission (OR = 3.26; 95% CI: 1.97 - 5.40), neonatal oxygen requirement (OR = 1.71; 95% CI: 1.00 - 2.90), hyperbilirubinemia (OR = 1.69; 95% CI: 1.07 - 2.66).
Ananth et al. (2003) & Demissie et al. (2002)* US, 1995-1997	269,287 twin pairs were divided to same sex and different sex (hence DZ) groups due to the lack of zygosity and chorionicity data	GA, SGA, sex; maternal age, education, illnesses (e.g., chronic hypertension, diabetes), parity, prenatal care utilization; placenta previa	Categorical variable grouped into predefined thresholds (<5%, 5–9%, … ≥40%)	20-40% depending on sex and size of the twins	Increasing BWD was associated with overall neonatal deaths (OR = 1.29; 95% CI: 0.99 - 1.68) among larger but not smaller twin of the same sex (this association was not found among twins of opposite sex). BWD ≥ 20% in same sex twin pairs (RR = 1.2; 95% CI: 1.1 - 1.4) and BWD ≥ 40% in different sex twin pairs (RR = 2.2; 95% CI: 1.7 - 2.8) was associated with risk for placental abruption.
Hartley et al. (2002)* Washington, US, 1987-1999	7,803 twin pairs, chorionicity and TTTS information was not available	GA, SGA	Categorical variable dichotomized into discordant vs. non-discordant	25%	BWD > 25% was associated with perinatal death (RR = 2.2; 95% CI: 1.5 - 3.1).
Redman et al. (2002) Michigan, US, 1995-2000	173 twin pairs (14% MC), GA > 23 weeks	GA, chorionicity	Categorical variable using thresholds of 90th, and 95th percentiles; and continuous variable (percent discordance)	30%	BWD > 30% was associated with cesarean section for non-reassuring fetal status, NICU admission, pH < 7.1 in umbilical artery, and 5-minute Apgar score < 7 (*p* <.0001).
Victoria et al. (2001) Missouri, US, 1993-1995	288 DC and 89 MC twins (5 with TTTS), GA > 24 weeks (N = 377 pairs)	GA, SGA, chorionicity maternal age, poor obstetric history	Categorical variable grouped into predefined thresholds (<5%, 5–25%, >25%)	25%	BWD > 25% was associated with increased odds for adverse neonatal outcomes like preterm birth < 36 weeks GA (OR = 5.05; 95% CI: 1.05 - 27.22), NICU stay of more than 10 days (OR = 3.28; 95% CI: 1.17 - 9.30) in severely discordant DC and MC twin pairs compared to severely discordant MC twin pairs.
Cooperstock et al. (2000) Missouri, US, 1978-1990	9,931 twin pairs, excluding pregnancies with fetal death (in either one twin)	GA, SGA, sex; maternal age (being a teenage mother), race, SES, parity, cigarette smoking	Categorical variable grouped into predefined thresholds (<30%, 30–40%, ≥40%)	30%	BWD > 30% was associated with preterm twin births at GA < 32 weeks (OR = 1.77; *p* < 0.03).

At the sample description the number of monozygotic (MZ) or dizygotic (DZ) twin pairs are shown. Wherever possible, the number of monochorionic (MC) and dichorionic (DC) pregnancies are indicated. Only percentages are indicated when chorionicity data was partially available. Most studies excluded monoamniotic twins, major congenital abnormalities (except for persistent ductus arteriosus in case of preterm birth), and twin-twin transfusion syndrome (TTTS); * shows where information on possible TTTS could not be obtained. Fetal death was defined as intrauterine death of the fetus ≥ 20 weeks of gestation, neonatal death was defined as death of the infant within 28 days after birth, whereas combined perinatal mortality included loss of twin(s) after 24 completed weeks or newborn weighting at least 500 g. Hospital death means death of newborn before discharge or within 120 days for infants still hospitalized. Preterm birth was defined as a birth that takes place before 37 weeks of pregnancy have been completed, if not otherwise specified by the study protocol (see details in the last column).

*Other abbreviations* BW: birth weight; BWD: birth weight discordance; CI: confidence interval; GA: gestational age; NICU: neonatal intensive care unit; NRDS: neonatal respiratory distress syndrome; OR: odds ratio; RR: relative risk; SGA: small for gestational age (less than the 10^th^ percentile); SES: socioeconomic status; twin A: the first baby delivered at birth

The largest epidemiological twin studies (using nationwide datasets) divided their analyses into same sex and different sex groups, because zygosity and chorionicity data was not available. They found BWD cut-off values of 15% for same sex twins and 30% for different sex (i.e., DZ and hence DC) twins conferring the greatest risks for fetal and neonatal death, and preterm birth [38]. Demissie et al. [40] showed that fetal mortality rates differed between the smaller and larger twins of the opposite sex (adjusted odds ratio > 2.7 from BWD > 20% among the smaller twins, but no consistent increase in the odds among the larger twins), whereas same sex twins had similar odds for fetal death (adjusted odds ratio > 1.7 from BWD > 20%). Later, Y. Chen et al. [41] included only discordant sex twins as proxy for chorionicity. Among sex-discordant premature DZ twins a cut-off value of 40% was associated with adverse neonatal outcomes (for details, see Table 1).

The earliest clinical studies included MC twins with TTTS, when blood flows from one of the twins to the other (creating donor and recipient twin). For example, Victoria et al. [42] found that severe BWD (> 25%) was more frequent in MC than DC twins, explained mostly by small placenta and umbilical cord abnormalities. Furthermore, Breathnach et al. [11] showed that the difference in BWD cut-off values associated with perinatal mortality and morbidity among DC and MC twins (18% *vs* 15%) was no longer observed when MC twins with TTTS were excluded from the analyses.

One of the first studies was conducted in the United States by Redman et al. [43], who identified a BWD greater than 30% as the threshold most predictive of adverse neonatal outcomes, including cesarean section, umbilical artery and neonatal intensive care unit admission. Later, a prospective study conducted in Ireland by Breathnach et al. [11] showed lower threshold; adverse neonatal outcome was increased with BWD exceeding 18%. In order to rule out other confounding factors, such as separate vs common placenta between DC and MC twins, Amaru et al. [44] controlled for chorionicity in their analyses, and reported that 20% BWD cut-off value was associated with cesarean delivery and adverse neonatal outcomes, like neonatal intensive care unit admission, oxygen requirement and hyperbilirubinemia. A Danish study found a threshold value of 15% in DC twins where low birth weight was associated with mortality [45]. Recently, in a Chinese cohort Ye et al. [46] reported cut-off values between 15–30% associated with different adverse neonatal outcomes.

In large twin cohorts of unknown chorionicity (ranging from 8,114–27,276 pairs), BWD thresholds varied between 20% and 40%. For example, threshold values between 30–40% were reported by an early study by Cooperstock et al. [9] who used a large US database from 1978 until 1990 to study the association between BWD and preterm birth. Using a population-based retrospective analysis of birth certificates and fetal/infant death certificates in the state of Washington, Hartley et al. [47] found BWD cut-off value of 25% indicative for perinatal and neonatal mortality. Boghossian et al. [8] examined BWD in premature, very low birth weight twins and found that most adverse outcomes including death before discharge from the hospital, necrotizing enterocolitis, severe retinopathy, and bronchopulmonary dysplasia, were more common among the smaller twins with the highest BWD (> 30%). The larger twins with the highest discordance level had increased odds of patent ductus arteriosus, moderate-to-severe cerebral palsy, and blindness. Even among term babies born between 36–40 weeks, Kim et al. [39] reported 20% BWD cut-off value associated with worse perinatal outcomes in a cohort of twins with vertex twin A delivered vaginally. Taken together, BWD cut-off values indicative for adverse perinatal outcomes are around 20–30%, their determination depends on the chorionicity, the sex of twins, the gestational age, and the presence of TTTS. Since the threshold value appears to be specific to each study, we indicated the utilized BWD cut-off for each epigenetic study in the result section Table 3.

**Table 3:** Epigenome-wide twin studies analysing DNA methylation levels and birth weight discordance.

Study	Twin cohort	Sample characteristics			DNA methylation assay			Covariates (in addition to cell ratios)	Findings
		N (twin pairs)	Age range	BWD cut-off	Tissue	CpG sites analyzed	Platform		
Casey et al., 2017	Quebec Newborn Twin Study (Canada)	52 MZ twin pairs (8 pairs with repeated samples after 3-6 months to assess stability)	15 - 17 years	No BWD cut-off value	Saliva	95,144 stable sites from 450k	Illumina Infinium Human Methylation 450 BeadChip Kit	- Birth weight - Sex - Family	BWD correlated with 426 CpG sites (46 collapsed CpG islands) at an unadjusted p < 0.005. No significant associations after correction for multiple comparisons.
Gordon et al., 2012	Peri/postnatal Epigenetic Twins Study (Australia)	22 MZ and 12 DZ twin pairs	At birth	No BWD cut-off value	HUVECs, CBMCs, placenta (only 7 pairs had all 3 tissues, 10 pairs had only 1 type)	~ 20,000 sites from 27k	Illumina Infinium Human Methylation 27 BeadChip Kit	- GA - Sex - Zygosity - Chorionicity	Few genes were associated with birth weight, e.g. apolipoprotein L domain containing 1 gene in HUVECs, sphingosine-1-phosphate receptor 1 in CBMCs.
He et al., 2016	MC pairs with selective FGR (China)	Discovery sample: 7 pairs; Replication sample: 12 twin pairs	At birth	BWD >20%	Placenta	~ 1.3 million sites in gene promoters	Bisulfite genomic sequencing	No covariates (no adjustment for cell composition was mentioned either)	4,605 sites (2,930 promoters) showed >20% methylation difference; 3 selected gene promoters (e.g., folate transporter and lecithin retinol acyltransferase from vitamin A metabolism) were validated in an independent sample of 12 twin pairs.
Marsit et al., 2013	Twin-twin transfusion syndrome children (USA)	4 male MC twin pairs and 6 sole survivors (5 females, 1 male)	5 months - 8 years	No BWD cut-off value	14 saliva and 11 whole blood	26,486 autosomal sites; LINE1 and ALU-Yb8 repetitive regions	Illumina Infinium Human Methylation 27 BeadChip Kit and pyrosequencing	- Age at sampling	Differentially methylated CpG sites were enriched at polycomb group target genes. Reduced methylation of the LINE1 repetitive element in blood samples among donors compared to recipients.
Martino et al., 2013	Peri/post-natal Epigenetic Twins Study (Australia)	10 MZ and 5 DZ twin pairs	At birth and 18 months	No BWD at age 1.5 year	Buccal cells (swabs)	330,155 sites from 450k	Illumina Infinium Human Methylation 450 BeadChip Kit	No covariates (did not report adjustment for cell types)	Methylation level at 99,198 sites (30.1%) changed over the first year of life, showing rapid changes in early postnatal development. No correlation was reported with birth weight.
Menni et al., 2013	TwinsUK	Discovery sample: 86 female MZ twin pairs; Replication sample: 175 pairs	32 - 80 years	BWD >750 g	Whole blood	24,641 autosomal sites from 27k	Illumina Infinium Human Methylation 27 BeadChip Kit	- Age at sampling - Sex - Body mass index - Zygosity	An ageing marker, glycosylated tryptophan level was associated with BWD, which was associated with methylation differences at 3 CpG sites, among which only the association at cg12757143 was replicated (in the promoter of the diphthamide biosynthesis 7 gene, coding for regulator of translation elongation factor 2).
Roifman et al., 2016	MC pairs with selective FGR (Canada)	8 MZ MC twin pairs	At birth	BWD >20%	Placenta	399,118 autosomal sites from 450k	Illumina Infinium Human Methylation 450 BeadChip Kit	- GA - Sex - Maternal age	8 genes showed > 10% methylation difference in their promoter region, e.g., leptin receptor and dienoyl-CoA reductase, which is important at unsaturated fat metabolism.
Shi et al., 2023	MC pairs with selective FGR (China)	Cohort 1: 12 MC twin pairs with FGR; Cohort 2: 15 selective FGR twins, 15 singleton FGR and 14 controls	At birth	BWD >25%	Placenta	850,000 sites	Illumina Infinium MethylationEPIC BeadChip; Pyro-sequencing in the validation cohort	No covariates (beside the adjustment for cell types) was mentioned	5,625 hypomethylated CpG sites and 452 hypermethylated CpG sites. Hypomethylation of cg00455178 in *CYP11A1* gene was validated with significantly higher mRNA expression level (but not protein level) changes.
Souren et al., 2013	East Flanders Prospective Twin Survey	17 female MC twin pairs	22 - 45 years	BWD >20%	Saliva	478,096 sites	Illumina Infinium Human Methylation 450 BeadChip Kit	- GA - Chorionicity	3,153 differently methylated CpG sites with nominal p < 0.01 and small methylation differences, which were not validated by deep bisulfite sequencing.
Chen et al., 2016; Tan et al., 2014	Danish Twin Registry (2 subgroups: young and old twins)	73 young MZ twin pairs and 77 old MZ twin pairs (from 150 pairs 28 selected for extreme BWD)	30 – 36 years and 57 - 74 years	No BWD cut-off in the whole dataset, BWD >25% for extreme cases	Whole blood	484,895 sites	Illumina Infinium Human Methylation 450 BeadChip Kit	- Age at sampling - Sex	No significant association at genome-wide level. The site-specific analyses highlighted cg20716652 (in the axon guidance molecule *RGMA*) in the whole sample, and cg05275595 (at the neutrophil granulocyte activating *CD177* gene) among extreme BWD twin pairs. Analysing genomic regions, the crystallin zeta gene was indicated, which was previously associated with resistin hormone level.
Tsai et al., 2015	Discovery sample: TwinsUK; Replication cohorts from Danish and Dutch twin registries	71 female MZ pairs from UK; 56 Danish MZ twin pairs (50% male); 89 Dutch MZ twin pairs (26% male)	34 - 78 years	BWD >20%	Whole blood	442,307 sites	Illumina Infinium Human Methylation 450 BeadChip Kit	- Age at sampling - Smoking - Alcohol consumption - Zygosity	One genome-wide significant association of BWD with cg12562232 in the insulin-like growth factor 1 receptor gene) was detected in the discovery sample (relatively healthy women), was replicated among older twin pairs (independent of sex).

In most EWAS analysing BWD the epigenome-wide data was obtained by the Infinium Human Methylation BeadChip array from Illumina (27k or 450k versions) and adjusted for heterogenous cell composition. Please note that a few studies used overlapping twin cohorts (their descriptions are included in the 2^nd^ column).

***Abbreviations:*** BWD: Birth weight discordance, CBMCs: cord blood mononuclear cells, GA: gestational age, DZ: dizygotic, HUVECs: human umbilical vein endothelial cells, MC: monochorionic, MZ: monozygotic; FGR: fetal growth restriction.

### Materials and methods for the scoping review of the epigenetic twin studies

We followed the PRISMA Guidelines and searched the PubMed and Ovid MEDLINE(R) databases up to June 19, 2025, for studies investigating the association between BWD and gene expression or DNA methylation, published in English. The following keywords were used in the PubMed database: “birth weight discordance”/ “discordant growth”/ “twin birth weight difference” & “gene expression”/ “DNA methylation”/ “DNA hydroxymethylation”. At the Ovid MEDLINE(R) we used “birth weight”/ “growth restriction” & “twin” & “gene expression”/ “DNA methylation”/ “DNA hydroxymethylation” keyword combinations in order to include wider range of reports. The automatic search was performed independently by two authors (DLW and ZN), who reviewed all abstracts independently. The inclusion criteria for our review were the followings: (i) published in peer-reviewed journal, (ii) examine BWD in human twins, (iii) focus on epigenetic mechanisms, like DNA methylation or gene expression. Disagreements about exclusion were resolved by discussion. Abstracts and full text of the potential papers were reviewed.

**Fig 2:**
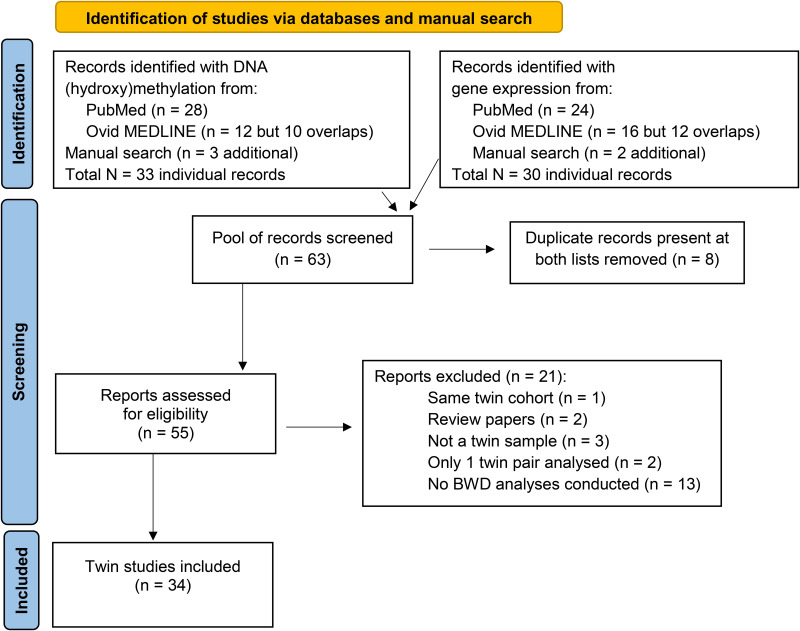
PRISMA flow diagram of included epigenetic and gene expression twin studies.

The PubMed search resulted in 36 papers with human data, the Ovid MEDLINE(R) search resulted in additional 8 papers on human samples (another 2 studies were conducted on animals). Finally, the automatic search was completed with a manual search from the reference lists of previous systematic reviews, meta-analyses, and the retrieved articles (adding one more paper). The combined search yielded 55 papers (after removing duplicates), from which 21 were excluded, because the report used the same twin cohort as in Gao et al. [48] paper (n = 1), or it did not include original research results (2 reviews), or not a twin sample (n = 3), or only one twin pair was examined as a comparison (n = 2), or there was no BWD analyses conducted with gene expression or DNA (hydroxy)methylation (13 papers). At the end, 34 independent papers were eligible for our review: 19 had gene expression, 21 had DNA methylation, and 2 had DNA hydroxymethylation measurements (8 of them were overlapping having both gene expression and (hydroxy)methylation data) (see Fig 2). The collected BWD associated genes were submitted to gene ontology (GO) analyses using DAVID (https://davidbioinformatics.nih.gov) [49,50]. Genes associated with BWD from placental tissues were analyzed separately from genes annotated to BWD associated CpG sites using cord blood or peripheral blood or saliva samples, see Supplementary xls. Older gene lists were reviewed, previously unannotated sites were annotated using the UCSC Genome Browser.

## Results

Among the 34 reviewed papers, 8 studies analyzed both gene expression and DNA methylation levels in newborn twin samples (see the parallel arrows showing the direction of differences at Table 2). First, we review the gene expression studies, since most of them used placental tissues, which carries out special functions during *in utero* development and the BWD associated genes might not function the same way in the child later in life.

**Table 2:** Epigenetic and gene expression differences leading to lower birth weight and birth weight discordance.

Gene symbol	Gene name	Function	Biological sample	DNA meth	mRNA level	Protein level	Study design and notes	Publication
*ADIPOQ*	adiponectin	Hormone secreted by adipocytes, regulating glucose levels and fatty acid metabolism	Cord blood	NA	NA	↓	14 discordant & 15 concordant twin pairs	Mazaki-Tovi et al., 2009
			Plasma	NA	NA	↓ #	89 young and 69 elderly twin pairs # sign. correlation among young twins	Storgaard et al., 2007
	*ADIPOR1*	adiponectin receptor 1	Plasma membrane receptors involved in fatty acid oxidation and glucose uptake, acting on muscle metabolism	Muscle tissue	NA	↓ *	89 young and 69 elderly twin pairs * sign. correlation among elderly twins	Storgaard et al., 2007
	*ADIPOR2*	adiponectin receptor 2						
*ADRB3*	adrenoreceptor beta 3	Catecholamine-induced receptor involved in the regulation of lipolysis and thermogenesis in the adipose tissues	HUVECs	NA	↑	NA	14 MZ twin pairs (8 MC, 6 DC pairs)	Gordon et al., 2011
*ANGPTL4*	angiopoietin-like 4	Hormone induced under hypoxia, regulates glucose and lipid metabolism, can act as an apoptosis survival factor for vascular endothelial cells	Placenta	hmC	↓	↓	4 selective FGR pairs and 4 normally developing MC twin pairs; sign. difference only among FGR pairs	Zhang et al., 2020
*COMT*	catechol-O-methyltransferase	Enzyme metabolizing catecholamines (e.g., dopamine, norepinephrine) and certain drugs	Buccal cells	— (no corr.)	NA	NA	12 MZ twin pairs	Mill et al., 2006
			Blood and saliva				20 MZ twin pairs treated for TTTS	Schreiner et al., 2011
*CDKN1C*	cyclin- dependent kinase inhibitor 1C	Strong inhibitor of several G1 cyclin & cyclin-dependent kinase complexes, negative regulator of cell proliferation	Placenta	NA	↓	↓	23 growth-discordant DZ twin pairs (no genetic test for zygosity, included different sex twins)	Yuen et al., 2018
*CRYZ*	crystallin zeta	NADPH-dependent oxidoreductase enzyme participates in one-electron transfer and detoxification of xenobiotics, interacts with AU-rich elements in the 3’-UTR of mRNA	Whole blood (adults)	↓	NA	NA	150 MZ twins	Chen et al., 2016
*DHCR24*	24-dehydro-cholesterol reductase	Oxidoreductase enzyme in the cholesterol biosynthesis (catalyzing the reduction of the delta-24 double bond of sterol intermediates)	Cord blood & whole blood of children, adults	↑	NA	NA	Meta-analysis of singletons’ EWAS	Küpers et al., 2019
*CYP11A*	cytochrome P450 family 11 subfamily A member 1	Monooxygenase enzyme involved in steroid hormone synthesis in mitochondria, catalyzing the conversion of cholesterol to pregnenolone	Placenta	↓*	↑*	NA	Cohort 1: 12 MC twin pairs with FGR; Cohort 2: 15 FGR twin pairs, 15 FGR singletons, 14 controls; * sign. difference among FGR twins and singletons	Shi et al., 2023
*EPO*	erythropoietin	Cytokine stimulating red blood cell production	Cord blood & placenta	NA	↑	↑	26 MC twin pairs (9 normal twin pairs, 17 twin pairs with FGR)	Chang et al., 2018
*FGF1*	fibroblast growth factor 1	Involved in embryonic development, organogenesis and cell growth	Placenta	NA	↓	NA	18 diamniotic MC twin pairs	Li et al., 2019
*GLI2*	GLI family zinc finger 2	Transcription factor binding to DNA through zinc finger motifs, activates patched Drosophila homolog (PTCH) gene expression, important during embryogenesis	Cord blood, blood from children up to 18 years	↑	NA	NA	Meta-analysis of singletons’ EWAS (using only child and adolescent samples)	Küpers et al., 2019
*GLUT3 (SLC2A3)*	glucose transporter 3 (solute carrier family 2 member 3)	Glucose transport across the blood-brain barrier, in the placenta, etc. (efficient transport even at low extracellular glucose concentration); galactose, mannose, maltose and dehydroascorbic acid import across plasma membrane	Placenta and cord blood	NA	↑*	NA	15 MC twin pairs with FGR (9 without and 6 with abnormal umbilical artery) *sign. difference only among twin pairs with umbilical abnormalities	Chang et al., 2021
			HUVECs	NA	↑	NA	14 MZ twin pairs (8 MC, 6 DC pairs)	Gordon et al., 2011
*GLUT4 (SLC2A4)*	insulin-sensitive glucose transporter	Insulin-regulated facilitative glucose transporter in the muscle and adipose tissues	Muscle tissue	NA	↓	NA	88 pairs of MZ & DZ twin pairs	Storgaard et al., 2006
*HERVWE1*	human endogenous retrovirus group W member 1	Involved in cytotrophoblasts’ fusion to form the syncytial layer of the placenta, promoting fetal growth through chorionic gonadotrophin and somatomammotropin pathways	Placenta	↓	↑	↑	21 discordant MZ twin pairs	Gao et al., 2012
*HIF1A*	hypoxia-inducible factor-1α	Master regulator of cellular and systemic homeostatic response to hypoxia, activates transcription of genes involved in energy metabolism, angiogenesis, apoptosis, etc.	Placenta	NA	↑*	NA	18 MC diamniotic twin pairs	Li et al., 2019
							15 MC twin pairs with FGR, *significant increase only in 6 FGR twins with umbilical abnormalities	Chang et al., 2022
*HLX*	H2.0 like homeobox	Transcription factor involved in organogenesis and lymphocyte differentiation	Placenta	NA	↓	↓	23 growth-discordant DZ twin pairs (no genetic test for zygosity, included different sex twins)	Yuen et al., 2018
*HOXC4*	homeobox C4	Transcription factor involved in morphogenesis	Cord blood & blood from children up to 18 years	↑	NA	NA	Meta-analysis of singletons’ EWAS (using only child and adolescent samples)	Küpers et al., 2019
*IGF2*	insulin like growth factor 2	A polypeptide growth factor involved in development and growth (coded by an imprinted gene, transcribed only from the paternal allele)	Cord blood, HUVECS & buccal cells	— (no corr.)		— (no diff.)	35 DZ and 56 MZ twin pairs	Ollikainen et al., 2010
			Blood from children at age 4				20 MZ twin pairs	Schreiner et al., 2014
*IGF2 BP1*	insulin like growth factor 2 mRNA binding protein 1	Regulates the translation of growth-related genes by binding to their mRNA and stabilizing the transcripts, such as insulin-like growth factor 2, beta-actin, beta-transducin repeat-containing protein	Blood samples	↓	NA	NA	17 adult MZ twin pairs	Córdova-Palomera et al., 2014
*LEP*	leptin	Hormone regulating food intake, body mass and reproductive function, plays a vital role in fetal growth	Placenta	↑ #	↑	↑	15 MC twin pairs (10 growth-discordant & 5 concordant) # measured among 8 discordant twin pairs	Schrey et al., 2013
*LEPROT*	leptin receptor overlapping transcript	Regulates expression of growth hormone receptor and leptin receptor	HUVECs	NA	↑	NA	14 MZ twin pairs (8 MC, 6 DC twin pairs)	Gordon et al., 2011
*LTA*	lymphotoxin alpha	Cytokine produced by lymphocytes, mediates inflammatory and antiviral responses, plays a role in apoptosis, involved in secondary lymphoid organ development	Cord blood, whole blood from children at age 7 & 17	↓	NA	NA	Singletons’ EWAS from the Avon Longitudinal Study of Parents and Children	Simpkin et al., 2015
*MAP4K2*	mitogen-activated protein kinase kinase kinase kinase 2	Serine/threonine protein kinase (can be activated by tumor necrosis factor), participating in B-cell differentiation	Cord blood, blood from children & adults	↓	NA	NA	Meta-analysis of singletons’ EWAS	Küpers et al., 2019
miR-210	miR-210-3p	Post-transcriptional regulation of genes involved in the hypoxia pathway and neuroinflammation	Placenta	NA	↑	NA	18 MC diamniotic twin pairs	Li et al., 2019
miR-29	miR-29b-3p	Post-transcriptional regulation of extracellular matrix genes (e.g., collagen, fibrillin, integrin and elastin), regulation of cell proliferation, differentiation	Placenta	NA	↓	NA	14 selective FGR twin pairs	Jiang et al., 2023
*NFIX*	nuclear factor IX	Transcription factor regulating intermediate progenitor cell generation in the developing cortex, neural differentiation in the hippocampus, and muscle regeneration	Cord blood & blood from children	↓	NA	NA	Singletons’ EWAS from the Avon Longitudinal Study of Parents and Children	Simpkin et al., 2015
*NOS3*	nitric oxide synthase 3	Producing nitric-oxide, involved in nervous system development and regulation of heart rate	Cord blood	NA	↑*	↑*	36 pairs of pre-mature twin neonates * sign. difference in twins compared to singletons	Dugmonits et al., 2016
*PPARD*	peroxisome proliferator-activated receptor delta	Integrator of transcriptional repression and nuclear receptor signaling, regulates lipid metabolism, differentiation, and epidermal cell proliferation	Muscle tissue	NA	↓	NA	88 young and elderly MZ & DZ twin pairs	Nilsson et al., 2007
*PPARGC1A*	peroxisome proliferator-activated receptor gamma coactivator 1-alpha	Transcriptional coactivator that regulates the genes involved in energy metabolism, regulates muscle fiber type determination	Blood and muscle tissue	NA	↓	NA	41 MZ twin pairs	Ling et al., 2004
*RB1*	retinoblastoma transcriptional corepressor 1	Negative regulator of the cell cycle, tumor suppressor, stabilizes constitutive heterochromatin	Placenta	NA	↑	↑	23 growth-discordant DZ twin pairs (no genetic test for zygosity, included different sex twins)	Yuen et al., 2018
*TGFB1*	transforming growth factor beta	Regulates cell proliferation, differentiation and growth, can modulate expression and activation of other growth factors including interferon gamma and tumor necrosis factor	Placenta	hmC	↑	↑	14 selective FGR twin pairs, protein levels were analyzed in 8 twin pairs	Zhang et al., 2020; Jiang et al., 2023

Gene expression and DNA (hydroxy)methylation differences are shown by arrows according to differences observed in the smaller twins compared to the larger twins. The associations between birth weight and DNA methylation, mRNA and protein levels observed in singleton birth cohorts are given to reflect differences resulting in lower birth weight.

*Abbreviations:* DC: dichorionic; DZ: dizygotic; EWAS: epigenome-wide association studies; FGR: fetal growth restriction; hmC: hydroxymethylcytosine; HUVECs: human umbilical vein endothelial cells; MC: monochorionic; MZ: monozygotic; NA: not assessed; no corr.: no correlation; sign.: significant

### Gene expression and birth weight discordance

The majority of gene expression studies selected *a priori* candidate genes implicated in metabolism or cardiovascular development or fetal growth (summarized in Table 2). For example, Gao et al. [48] used placental samples to examine the expression of the human endogenous retrovirus group W member 1, envelope gene (*HERVWE1*) known to be highly expressed in syncytiotrophoblasts when generating the syncytial layer of the placenta. In addition, HERVWE1 is involved in several endocrine hormone pathways, such as the human chorionic gonadotrophin and somatomammotropin pathways, that promote fetal growth. In addition to 56 MZ DC twin pairs, they collected data from 10 singleton pregnancies (for comparisons’ reason, none of them had growth restriction, and all of them were delivered by cesarean section between 30–36 gestational weeks). Significant differences were observed only among the 21 discordant twin pairs with at least 20% BWD: *HERVWE1* mRNA and protein levels were increased in the smaller twins compared to the larger twins, with an accompanying decrease in DNA methylation level at the gene’s promoter region. Furthermore, the *HERVWE1* transcript level was negatively correlated with birth weight in all the groups, which was attributed to the unique characteristics of the study population, consisting of discordant (but not FGR) twins with birth weights within the normal range.

Some researchers examined twin pregnancies with selective FGR, i.e., when the smaller twin’s birth weight is less than 10^th^ percentile in the third trimester, with inter-twin BWD > 20–25%. For example, Schrey et al. [51] investigated 88 angiogenesis-related genes in placental samples of 10 severely growth-discordant MC twins (BWD ≥ 20%, without TTTS) and 5 growth-concordant MC twins. They found 3 genes showing mRNA expression differences between the smaller and larger twins, and confirmed at the protein level that leptin expression, which regulates food intake and plays a vital role in fetal growth, was increased in the smaller twins of the discordant growth group. The DNA methylation analysis of the leptin gene promoter showed higher methylation in the smaller twins, potentially resulting in a reduced binding of a repressor type of transcription factor at this regulatory region.

Similarly analyzing twin pairs with selective FGR, Chang et al. [52] measured cord blood erythropoietin level and placental erythropoietin gene expression in MC twins delivered through elective cesarean section (9 normal pairs, 9 pairs with selective FGR but without umbilical artery abnormalities, 8 pairs with FGR and with artery abnormalities), and calculated ratio of erythropoietin level of the smaller/ larger twin within each twin pair. They found a good correlation between fetal plasma erythropoietin and placental expression ratios in the whole sample. Twins with FGR and umbilical artery abnormalities had the highest plasma erythropoietin level (compared to the other two groups), possibly compensating for the suboptimal circulation, increasing erythropoiesis to enhance the oxygen-carrying capacity of their blood. The smaller twins of pregnancies with umbilical artery abnormalities had two times higher cord plasma erythropoietin level and placental expression compared to their co-twins, who had significantly elevated erythropoietin level compared to the other groups (they also had significantly shorter gestation period and smaller birth weight compared to the other twins). The same group later examined placental glucose transporters’ expression. Only twins with FGR and umbilical artery abnormalities differed from the others at the glucose transporter 3 (*GLUT3/SLC2A3*) level [53]. Using placental mesenchymal stem cells derived from MC pregnancies with selective FGR, Chang et al. [54] finally showed that the group of growth-restricted fetuses had lower capacity to increase the placental glucose transporter expression under hypoxic conditions compared to their co-twins appropriate for gestational age (see glucose transport mechanism in the placenta at Fig 1). Since GLUT3 has a lower Km compared to GLUT1, which enables efficient glucose transport under low extracellular glucose concentration, these compensatory mechanisms are physiological [for more details see reviews by 55, 56].

Investigating expression level of *HLX* homeobox gene coding for a transcription factor and its downstream target genes (e.g., retinoblastoma and cyclin dependent kinase inhibitors), Yuen et al. [57] reported decreased expression of *HLX* and cyclin dependent kinase inhibitor 1C (*CDKN1C*) in the FGR twins’ placental samples compared to the normal weight co-twins’ placental parts at 23 DZ twin pregnancies. Although the selection of twin pregnancies was not optimal, as sex and genetic effects cannot be excluded, the differential expression was confirmed at both the mRNA and protein levels, reflecting delayed fetal growth.

Another type of regulation was studied by Li et al. [58], analysing microRNAs in 18 placenta sections of diamniotic MC twins (excluding TTTS, chromosomal abnormalities, and maternal diseases). Increased expression of miR-210-3p was found in the FGR group, which can lead to decreased expression of fibroblast growth factor 1. Their *in vitro* experiments supported the following model: under hypoxic conditions, level of hypoxia-inducible factor-1α (*HIF1A*) is increased, causing an increased miR-210-3p expression, which in turns inhibits fibroblast growth factor 1 gene expression. Altogether, this leads to impaired proliferation of the trophoblast cell line (see regulatory process at Figure 1).

*In vivo* assays were also used in twin studies to decipher birth weight-related epigenetic mechanisms leading to health problems later in life, since metabolic diseases, such as diabetes mellitus are often linked to prenatal risk factors [59]. In a series of papers, almost a hundred healthy, full-term twin pairs (born 40 ± 3 gestational weeks) from the Danish Twin Register were analysed for metabolic markers in muscle biopsy samples taken before and during insulin infusion (testing their insulin sensitivity). Among the 41 MZ twin pairs with gene expression data, birth weight was positively associated with the insulin-stimulated expression of *PPARGC1A* (peroxisome proliferator-activated receptor gamma coactivator 1-alpha) and glucose oxidation rate [60]. Significant positive association was found between birth weight and both baseline and insulin-stimulated glucose transporter 4 level measured in the muscle [61]. Later, using young (aged 25–32 years) and elderly (aged 58–66 years) twin pairs, they showed similar positive associations between birth weight and peroxisome proliferator-activated receptor delta muscle expression [62]. In addition, plasma samples were analysed for adiponectin, which is an insulin-sensitizing adipokine acting on muscle metabolism [63]. By adjusting for intra–twin pair dependency and assessing correlations of within–pair differences, the analyses revealed a nongenetic effect of birth weight on both plasma adiponectin levels and muscle expression of adiponectin receptor 1 among MZ twins. Interestingly, birth weight was also associated with *in vivo* measures of glucose metabolism in these twins, highlighting the fetal programming effects on glucose homeostasis, and their possible roles in the pathogenesis of type 2 diabetes later in adulthood [64] (for more details see Table 2).

Analysing the role of oxidative stress in delayed fetal development and subsequently low birth weight, Dugmonits et al. [65] measured the levels of reactive oxygen species in red blood cells from twin and singleton neonates. Higher levels of hydrogen peroxide and nitrate, and consequently elevated protein and lipid damages were reported in twin neonates compared to singletons. Expression levels of nitric oxide synthase and other enzymes of the antioxidant defence system (including catalase, superoxide dismutases and haemoxygenases) showed that premature twins with lower birth weights had the lowest antioxidant capacity.

One of the first genome-wide studies analysing associations between BWD and gene expression without *a priori* hypotheses used multiple tissue types from the Peri/postnatal Epigenetic Twins Study. Gordon et al. [66] showed significant negative correlations between birth weight and expression of genes related to metabolism and cardiovascular function. They found 41 genes significantly linked to birth weight in human umbilical vein endothelial cells (HUVECs) analysed from 14 twin pairs. Comparing cord blood mononuclear cells, only DC twins showed differential expression at 342 genes, highlighting the possibility that MC twins, who share the same placenta, have lower gene expression difference. They highlighted three genes involved in fetoplacental growth and metabolism, which expression in HUVECs was negatively correlated with birth weight: adrenoreceptor beta 3, glucose transporter 3, and the leptin receptor overlapping transcript (*LEPROT*), which has an important regulatory role in the cell surface expression of growth hormone and leptin receptors (for more details, see Table 2). These results suggest compensatory mechanisms at the expression of genes involved in fetoplacental growth and supplying energy.

Recently, Zhang et al. [67] compared placental mRNA levels and DNA hydroxymethylation patterns of 4 twin pairs with selective FGR to 4 normally developing MC twin pairs. Their mRNA-sequencing data showed 81 up-regulated and 614 down-regulated genes in the placental shares of the FGR group compared to the co-twins. Analysis of the overlapping genes with differential mRNA expression and DNA hydroxymethylation levels pointed to the involvement of angiopoietin-like 4 (*ANGPTL4*) gene. Based on knockdown experiments conducted *in vitro*, the authors suggested a model in which hypoxia through the HIF1A pathway changed the *ANGPTL4* gene promoter methylation pattern, leading to decreased *ANGPTL4* level [67]. Further exploring the molecular process of this regulatory pathway, Jiang et al. [68] measured the TET enzyme family (proposed to be the main catalysators of the active DNA demethylation processes), as well as their activators, inhibitors, and downstream targets in placental samples of 14 pregnancies with selective FGR (without TTTS or other reversed arterial perfusion). They identified a downregulated microRNA (miR-29b-3p) causing increased TET2 enzyme activity and subsequently increased transforming growth factor beta 1 (TGFB1) level in the FGR samples. Since TGFB1 is an important regulator of cell proliferation and differentiation, the authors highlighted the role of miR-29b-3p-TET2-TGFB1-smad axis leading to reduced trophoblast invasion and to increased apoptosis in growth retardation.

Altogether, using neonatal cells from cord blood or placental tissues at birth, gene expression studies provided evidence that prenatal environment can affect expression of genes involved in fetal growth (e.g., *LEPROT, HERVWE1, TGFB1*), oxygen and glucose delivery (e.g., erythropoietin, glucose transporters), and hypoxia-signaling cascade (e.g., HIF1A). The genome-wide and candidate gene expression studies indicated upregulated genes involved in maintaining optimal glucose and oxygen concentration during *in utero* development, which might be still sub-optimal leading to lower birth weight. However, it has to be noted that most reports did not have replication cohort and used relatively small sample size.

### DNA methylation and birth weight discordance

Except for a few studies analysing placental tissues, most research groups investigated BWD associated DNA methylation patterns in cord blood or later in life using peripheral blood or saliva samples. The most recent analyses used sequencing or the Illumina EPIC array with over 850 thousands of CpG sites, whereas the majority of published data is based on the Illumina 450k array (see details in Table 3). Researchers also started to examine another covalent modification of the genomic DNA, the hydroxymethylation, which can serve as a marker for active demethylation, hence gene activating processes, see the above-mentioned study by Zhang et al. [67] where they highlighted that the aberrant hydroxymethylation pattern at the *ANGPTL4* gene resulted reduced protein expression in the smaller placental shares, leading to reduced trophoblast functions, and subsequent suboptimal growth.

The earliest epigenetic twin studies used tissues at birth and focused on FGR or other medical problems. For example, Bamforth et al. [69] analysed X-inactivation patterns in chorion, amnion, and cord samples of 79 female twin pairs, specifically selected for BWD, discordance for congenital anomalies, TTTS, but no correlation was seen with BWD or with any other clinical outcome. The latest studies rely on more sophisticated methods, which allow detection of smaller differences in DNA (hydroxy)methylation levels, either via candidate gene approach or at the epigenome level. The majority of studies selecting *a priori* candidate genes analysed imprinted regions, e.g., the maternally imprinted insulin-like growth factor 2 gene (*IGF2*) and the paternally imprinted *H19* gene (coding for a tumor-suppressor long non-coding RNA), which have a common imprinting center at the 11p15 chromosomal region [70]. Once the specific gene is methylated by the imprinting process, it is inactivated on either the maternal or paternal chromosome in a parent-of-origin effect, e.g., the growth regulator *IGF2* gene is expressed only from the paternal chromosome, because it is imprinted, hence inactivated on the maternal chromosome.

### Candidate gene approach

The earliest epigenetic studies measured DNA methylation level in the regulatory region around the transcriptional start site of only one chosen candidate gene. For example, Mill et al. [71] analysed the promoter region of the catechol-O-methyltransferase (*COMT*) gene using buccal cells of 5-year-old healthy MZ twin pairs with high (27–40%) BWD. This gene was selected as a well-studied candidate in a chromosomal region linked to schizophrenia, with evidence that its interaction with early-life factors like low birth weight may affect psychopathology risk, making it relevant for studying methylation differences in birth weight–discordant MZ twins. The methylation levels of the studied CpG sites showed wide variation among the 12 MZ twin pairs, and there was no correlation with birth weight. Later, Schreiner et al. [72] examined 20 MZ twins at age 3–5 years, who were treated for TTTS before the 27^th^ gestational week. They analysed peripheral blood and saliva samples from the twin pairs and unrelated control children and reported high intra-twin-pair correlation in the methylation level of the *COMT* promoter region in both biological sample types. Importantly, they could not detect any correlation between methylation differences and pre- or postnatal growth patterns.

It is important to mention that the early epigenome wide studies on BWD did not control for cell heterogeneity, which can cause substantial differences in tissue-specific methylation patterns both in blood-related and oral samples. This is especially true at alternative promoter regions, such as the one regulating the soluble *COMT* isoform studied by Mill et al. [71] and Schreiner et al. [72]. In this aspect, tissue samples with predominantly one cell type are more comparable. Using placental samples (from which blood was carefully removed by washing steps), Gao et al. [48] reported reduced DNA methylation at the *HERVWE1* gene promoter in the lower birth weight twins within the discordant pairs, compared to their larger siblings, whose methylation values were similar to that of singleton babies born at similar gestational age via cesarean section.

Later on, the majority of epigenetic twin studies using the candidate gene approach focused on the imprinted region on chromosome 11p15 containing the *IGF2* and *H19* genes. Various biological samples from individuals of different ages were used. The first such report analysed neonatal and placental samples collected at birth from the Australian Peri/postnatal Epigenetic Twins Study. They examined multiple tissues, such as cord blood (separated to mononuclear cells and granulocytes), HUVECs and buccal cells from 35 DZ and 56 MZ twins, among whom 19 were DC [73]. Substantial differences due to tissue specific methylation patterns were observed, but no strong correlation could be detected with BWD (Spearman correlation coefficients ranged from −0.45 to 0.58). Analysing 4-year-old children previously treated for TTTS, Schreiner et al. [74] could observe only one weak association between BWD and slight increase in methylation at the *IGF2* gene region, more pronounced in saliva than in blood (with Spearman correlation coefficient 0.51). However, no difference was found in the serum IGF2 level between donors and recipients either at birth or at age 4. Furthermore, no correlation was observed between IGF2 serum levels and methylation values using the intra-twin differences. After widening the candidate regions and including IGF2-related developmental genes, such as *IGF2 BP1–3* (IGF2-binding proteins 1–3), Córdova-Palomera et al. [75] found a positive association between birth weight and DNA methylation level of two CpG sites in the *IGF2 BP1* gene in peripheral blood samples of 17 healthy adult MZ twin pairs (among the 248 preselected sites of the IGF2 pathway). Each kilogram increase in birth weight corresponded to approximately 8.3% increase in DNA methylation level of the growth-regulator *IGF2 BP1* gene, after controlling for sex, gestational age, and age at sampling (which ranged from 22 to 56 years). Since this RNA binding protein stabilizes *IGF2* transcripts, its reduced expression can result in lower IGF2 level (see more details in Table 2). It is important to mention that the rest of the studied sites had negligible inter-individual variation (less than 5% of methylation differences), hence they were not informative to reflect non-shared environmental effects.

Taken together, candidate gene twin studies resulted mostly in negative findings, either because of the low variability in the methylation level at the targeted sites or the less sensitive methods used. This is expected because methylation differences are often less than 10% in easily available, heterogeneous samples like whole blood or saliva.

### Epigenome-wide association studies (EWAS)

The first EWAS of BWD used samples collected at the Peri/postnatal Epigenetic Twins Study. Gordon et al. [76] compared different tissues obtained at birth from MZ and DZ twin pairs, and found a few significant associations between DNA methylation levels at genes involved in lipid metabolism and birth weight of twins (for details see Table 3). Martino et al. [77] compared DNA methylation patterns of buccal epithelial cells collected at birth and 18 months later with the wide-spread methylation array type from Illumina (450k BeadChip array). DNA methylation levels changed at many CpG sites over the first 1.5 year of life, but the changes were not associated with BWD. It should be noted that these pilot measurements regarded the biological samples (relatively) homogenous and no adjustment for cell composition was mentioned at their analyses. Their main aim was to estimate the genetic and non-shared environmental effects in the framework of the DOHAD theory by studying MZ and DZ twins. Later on, epigenetic twin studies included only MZ twins, with sample sizes ranging from 17 to 150 pairs (see details in Table 3).

In their landmark paper using saliva samples from female MC twin pairs, Souren et al. [78] showed that heterogenous cell composition was driving the largest methylation differences in their epigenome-wide dataset obtained with the Illumina 450k array. After controlling for the buccal epithelial cell ratio (and removing the outlier twin pair, who were the only heavy smokers in the cohort), they showed that BWD was associated with more than 3,000 CpG sites, but only 45 of them had methylation differences > 5%, which was regarded as the threshold for technical variation (these 45 CpG sites are listed in the Supplementary file). Importantly, they applied a different technique, the deep bisulfite sequencing to confirm methylation changes at 8 selected CpG sites. Although the methylation values showed good correlation at 5 out of 8 loci between the two methods, the sequencing data produced much less variation (the 5–7% methylation differences between the discordant twins reduced to ≤ 1% differences), doubting the biological relevance of the studied CpG sites. In order to support their negative findings, DNA methylation levels at repetitive sequences were also measured, resulting in non-significant differences between the heavy and light co-twin groups.

Using saliva samples collected from young adults, later twin EWAS could not detect any significant association either between BWD and DNA methylation patterns [14]. Similarly, no significant associations were observed between pre-selected epigenetic markers and birth weight in a sample of 1,040 twins using buccal cells collected at age 6–13 [79]. Interestingly, using blood samples of adult twins from the Netherlands Twin Register (collected at age 18–79) weak association was detected between birth weight and DNA methylation score calculated from Illumina array data of 934 CpG sites indicated by the meta-analysis of Küpers et al. [18]. However, this combined DNA methylation score explained only 0.39% of the variance (whereas polygenic scores explained 1.52% of the variance) in birth weight [79]. For reasons of comparisons, we have to note that much higher variance was explained by combined DNA methylation scores at prenatal maternal smoking (16.9%), and body mass index (6.4%) in the same adult dataset.

A few research groups selected extreme cases of BWD in order to find differentially methylated regions in the genome. The first pilot study examined a special sample of children, survivors of TTTS, treated with laser surgery between 16–24 weeks of gestation [80]. Only subtle changes (~ 5% differences) were detected at specific CpG sites from blood and saliva samples between donors and recipients, but they were more likely situated in genes controlled by polycomb group proteins, highlighting developmentally important regions. Subsequent epigenome-wide studies examined placental tissues from twin pregnancies with selective FGR. Using the most common DNA methylation array, a Canadian group detected at least 10% within-pair methylation differences at 8 genes [81] (see Table 3 for study cohort description).

Applying a similar twin study design of TTTS cases, in-depth analyses by He et al. [82] found 2,930 differentially methylated promoter regions. Selecting four genes related to specific biological functions (e.g., involved in cell migration, RNA binding, vitamin A metabolism, and folate transportation), methylation differences at three target regions were validated in an independent sample of 12 twin pairs. However, DNA methylation differences at these regulatory regions were not correlated with gene expression differences in the placental tissues from 11 pairs. Compared to normally developing co-twins, the placental shares of growth-retarded twins showed lower general DNA methylation and hydroxymethylation levels (measured by mass spectrometry), supporting the epigenetic differences between discordant twins.

Measuring placental samples of MC growth-restricted fetuses compared the their co-twins, Shi et al. [83] found hundreds of CpG sites differentially methylated. Their analysis of affected pathways revealed dysregulation primarily in steroid hormone biosynthesis, metabolism, cell adhesion, and immune response. Importantly, in an integrative analysis of the promoter methylome of FGR placentas, hypomethylation at the *CYP11A1* gene was highlighted when they compared their own dataset with previously published ones [81,82]. The *CYP11A1* gene codes for a cytochrome *P450* monooxygenase involved in the synthesis of cholesterol, steroids, including vitamin D. It catalyzes the conversion of cholesterol to pregnenolone, the first, rate-limiting step in the synthesis of the steroid hormones. Therefore, up-regulation of this enzyme in placenta can potentially disturb progesterone synthesis and can even trigger oxidative stress, leading to growth restriction.

Most of the EWAS used the Illumina 450k methylation array with easily accessible samples, e.g., saliva [78,84] or whole blood samples [85–88]. Only one of them reported a differentially methylated CpG site at the genome-wide significance level using a female MZ twin sample with BWD > 20% selected from the TwinsUK cohort. Interestingly, this site in the insulin-like growth factor 1 receptor (*IGF1R*) gene could be detected only with quantitative analysis using BWD as a continuous variable [88]. Using the same TwinsUK sample in their ageing-related metabolic analyses, Menni et al. [87] detected significant associations between an ageing marker, the glycosylated tryptophan level and BWD at 3 genes (diphthamide biosynthesis 7, endothelin 2, galactosidase beta 1 like 3), among which one was replicated in their combined dataset of 522 individuals. Association between glycosylated tryptophan metabolite and DNA methylation level at cg12757143 in the promoter region of the diphthamide biosynthesis 7 gene pointed to the involvement elongation factor 2 regulation, which is important not only in protein synthesis but also in the regulation of cell proliferation and migration. Importantly, similar associations could be detected in other, independent twin samples (see Table 3 for details).

Using a relatively large (and not preselected) twin cohort, Tan et al. [85] conducted EWAS on 150 MZ pairs using both quantitative analyses (with BWD as a continuous variable) and qualitative analyses (comparing the larger and smaller co-twins), but could not detect any genome-wide significant associations. In the same dataset of the Danish twin cohort, M. Chen et al. [89] searched for differentially methylated gene regions. One BWD-associated chromosomal region (1p31) could be detected in their quantitative analyses, involving a metabolism-related gene, the crystallin zeta gene coding for a NADPH-dependent quinone reductase, which was previously associated with resisting hormone levels in a genome-wide association study [90], and subsequently linked with insulin resistance (an important metabolic marker of prediabetes, showing the reduced sensitivity to insulin in the fat and muscle tissues). However, we have to note that using the same Danish twin cohort, Frost et al. [91] found no evidence for a detrimental effect of low birth weight on glucose metabolism in adulthood (indexed by plasma glucose, insulin, and other hormones’ levels).

In order to reveal biologically relevant genes or pathways affected by BWD, we run GO term analysis of BWD associated genes by the DAVID functional annotation (available as a NIH tool). CpG sites indicated in placental samples were analyzed separately since long-standing epigenetic changes cannot be measured in this tissue which functions only during *in utero* development. Also, Shi et al. [83] already analyzed the overlapping genes of the FGR studies using placenta samples (published by Gordon et al., [76]; He et al., [82]; Roifman et al., [81]). The gene lists and detailed results can be found in the Supplementary file. The top molecular processes of placental BWD associated gene were linked to nervous system development and to transcription regulation (affecting RNA polymerase II). There were less genes indicated from cord blood, peripheral blood or saliva samples collected later in life from twins, therefore GO term analysis indicated only nominally significant processes (with p-values < 0.05 but FDR corrected p-values > 0.1), highlighting phosphatidylinositol 3-kinase/protein kinase B signal transduction, axon guidance and other cell proliferation processes.

In addition, we also checked for overlapping genes associated with birth weight as a continuous variable in singleton cohorts and with BWD by comparing heavy and light co-twins in twin pregnancies. Only a few overlapping genes were detected, such as the PPARG coactivator 1 beta (*PPARGC1B*) involved in fat oxidation and regulation of energy expenditure, or the RAS p21 protein activator 3 (*RASA3*) participating in one of the important pathways regulating cell growth and division.

## Discussion

The present review aimed to summarize and synthesize the state of research about epigenetic modifications associated with BWD in twin pregnancies. Overall, studies investigating gene expression in twins discordant for birth weight suggested that altered placental gene regulation plays a key role. Specifically, changes in pathways related to growth, angiogenesis, glucose transport, hypoxia response, and metabolism have been associated with lower birth weight. These alterations may contribute to an increased long-term risk for metabolic disorders, such as type 2 diabetes. In terms of epigenetic studies, early research focusing on candidate genes largely yielded negative or inconclusive results. This may be due to limited methylation variability at the targeted sites or the use of less sensitive methods. In contrast, more recent epigenome-wide studies, particularly those using placental samples, have identified differentially methylated regions associated with BWD. These regions frequently involve genes implicated in biological processes relevant to fetal development, such as growth regulation, metabolic pathways, and cellular signaling.

We have to mention that birth weight, as other complex traits, has heritable genetic components, but it is predominantly driven by gestational age. After controlling for gestational age, genetic and unique environmental effects accounted for roughly equal shares of the variation in birth weight (20–25%), although there were some cultural-geographical differences [92]. Therefore, researchers tried to explore these unique environmental effects and their potential underlying biological mechanisms. As in many complex, polygenic traits, birth weight is controlled by hundreds of genetic variants and possibly similarly high numbers of epigenetic modifications, each contributing a small percentage to the variance. Using large-scale genome-wide datasets, researchers could distinguish maternal factors (driven by maternal genome) from the offspring’s genetic impact on birth weight [93]. These analyses highlighted the importance of maternal glucose level and fetal circulation in fetal growth. Confirming the relevance and importance of genetic variants, later analyses including thousands of study participants with extreme (high and low) birth weight were needed [see for example 94].

Although candidate gene analyses supported the involvement of leptin signaling pathway and glucose transport in BWD, it should be noted that most expression studies used less than 30 samples per group, therefore, they can be regarded as small-scale pilot studies. Much bigger sample sizes were available at the DNA methylation studies, but the results obtained from recent EWAS are still inconsistent. It might be because of the weaker functional relevance, since DNA methylation changes by themselves do not necessarily reflect gene expression changes, as it has been demonstrated at the imprinted *H19* or *IGF2* genes [95]. Importantly, most studies could not detect any significant differences in DNA methylation patterns between the heavy and light co-twins using categorical analyses. The most promising findings were obtained from blood samples of MZ twins with BWD > 20% using quantitative analysis [88,89]. Epigenetic profiles of saliva samples showed much more variability [78].

Reasons for the inconsistent findings may lie in the study design. First, the age of sampling, i.e., the time period at which methylation measurements were taken, differs from study to another. For example, DNA methylation was examined after delivery, during adolescence or in adulthood (see Table 3). The results are therefore not always comparable, since DNA methylation is not fixed at many CpG sites over the life span, although there are certain sites with stable methylation pattern, as shown by longitudinal studies with multiple timepoints [e.g., 96, 21]. Second, DNA methylation level was measured mostly only once, and from only one tissue type, using Illumina arrays which have preselected CpG sites of the human genome. Third, potential differences may also stem from the type of tissues used in these studies (e.g., saliva, blood, neonatal tissues) since cellular heterogeneity was not controlled in the earlier studies and each cell type has its unique epigenetic landscape that likely reflects its specific function and response to environmental exposures. It should also be noted that most of the EWAS included all ~ 480 thousand CpG sites from the 450k methylation array. However, the majority of CpG sites on Illumina arrays show little variability between individuals. This likely had a significant impact on the statistical analyses. For this reason, the number of analyzed CpG sites is indicated in Table 3.

Only two studies employed a continuous analysis of BWD, despite its potential to capture more nuanced associations across the full range of discordance values. Instead, most research opted for categorical approaches, using predefined thresholds (e.g., ≥ 15%, ≥ 20%, ≥ 30%) to classify twin pairs into discordant and non-discordant groups. While such thresholds offer clinical simplicity and facilitate group comparisons [39,46], they may overlook subtler gradations in risk that could be clinically meaningful. Continuous modeling—such as using BWD as a continuous predictor in spline regression or ROC curve analysis—can provide a more precise understanding of how increasing levels of discordance relate to adverse outcomes [97]. The limited use of this method highlights an important gap in the literature and suggests the need for more refined statistical approaches in future twin studies.

Epigenetic regulation of growth-related genes in the offspring may be a relevant mechanism through which adverse prenatal environment is associated with low birth weight. The literature also shows similar trends in studies of singletons. Most researchers that examined singletons showed that birth weight (adjusted or not for gestational age) was associated with DNA methylation patterns at birth or later in life at certain CpG sites involved in growth or in embryonic development [18]. More recent studies did not replicate the previously identified genes, however, they used much smaller sample sizes, hence they may have been underpowered to detect few percentages of epigenetic difference between low and high birth weight infants [98,99]. Comparing epigenetic and gene expression profiles between twins and singletons can reveal both shared and unique regulatory patterns, as twin studies can highlight specific mechanisms related to intrauterine competition and growth asymmetry that may be obscured in singleton cohorts. Future research should consider including multiple layers of biological factors, genome-wide analyses of gene expression and DNA methylation levels should supplement the already advanced analyses of genetic variants. Integrating findings across biological variables may enhance our understanding of the developmental origins of health and disease and guide tailored interventions for at-risk newborns. Taken together, this review showed how DNA methylation patterns associated with BWD are characterized by variability in methodology (e.g., sampling time, tissue type and DNA methylation measure used), likely contributing to the inconsistency of results reported.

We can potentially expect a couple of reliable findings at the genome-wide significance level in the near future, as there are many large twin studies with EWAS data using peripheral blood samples collected from young adults (age 18–36 years), such as the UK-based E-Risk Twin Study (N = 1629), the Finnish Twin Cohorts (N = 757), and Netherlands Twin Register (N = 2059) (see the discovery and replication samples of a recent meta-analysis by van Dongen et al., [100]. These twin samples are part of the ACTION project (Aggression in Children: Unraveling gene-environment interplay to inform Treatment and Intervention strategies) which is now in the analytical phase. Newer, more sophisticated analyses could detect important epigenetic alterations from early developmental stages, which can stay stable in our genome and can serve as useful epigenetic markers later in life (at the potential disease prognostic stage). Prospective studies with reliable, detailed information about perinatal factors are needed to highlight individual DNA methylation sites with small effects.

Finally, we would like to note some gaps in the literature, which can be addressed by future studies. For example, researchers could examine several candidate genes with well-established involvement in fetal growth (e.g., *IGF2*, *LEPROT, ADRB3*, *GLUT3*) in two or more tissues and developmental stages (e.g., in placenta, cord blood or umbilical vein cells, and in child and adult blood or saliva samples), which can help finding stable epigenetic modifications associated with DNA methylation differences between the co-twins. Further, most studies conducted to date have been cross-sectional in design and relied on single epigenetic measures using samples collected only once at different time points, like delivery, early childhood, adolescence or adulthood. Longitudinal studies with several data collection points for epigenetic measures would be useful to comprehensively and prospectively investigate the long-term effects of prenatal environmental exposures indicated by BWD. In addition, few of the association studies used extreme BWD pairs of twins (i.e., BWD > 30–40%). Our understanding may be improved by including twins with wide range of BWD.

Lastly, one major limitation across existing studies is the lack of conclusive data on differential gene expression in the context of BWD, which may stem from methodological constraints. These include, for example, small sample sizes, variability in the tissues analyzed, and differences in gestational age at sampling [76,78]. Such factors can influence gene expression independently and reduce comparability across studies. Additionally, the absence of standardized thresholds for defining BWD further complicates interpretation and integration of findings.

## Conclusion

There is no universally accepted BWD threshold predicting adverse outcomes, as cut-offs—typically between 15% and 40%—vary widely depending on chorionicity, gestational age, study design, and whether BWD is analyzed categorically or continuously. This review found several studies which showed that prenatal environment experienced *in utero* by twins can be associated with BWD through altered gene expression, particularly at genes involved in pathways affecting cell proliferation, development, which in turn can affect birth weight. However, studies generally have small sample sizes and did not include replication cohorts. Larger size twin studies are available examining BWD and DNA methylation patterns, but have not provided consistent evidence yet, possibly due to the small methylation differences in tissue samples with heterogenous cell compositions. In order to reveal robust associations, future epigenome‐wide analyses should pay attention to several confounding parameters to be controlled for (e.g., smoking), and include sample sizes with sufficient power to detect a few percentages of methylation differences.

## Supporting information

S1 File Top ten gene ontology terms of BWD associated genes. (XLSX)
